# Transcriptome-wide association study identifies genes associated with bladder cancer risk

**DOI:** 10.1038/s41598-025-85565-3

**Published:** 2025-01-09

**Authors:** Siting Li, Jiang Gui, Margaret R. Karagas, Michael N. Passarelli

**Affiliations:** 1 Department of Biomedical Data Science, Geisel School of Medicine at Dartmouth, Hanover, NH USA; 2 Department of Epidemiology, Geisel School of Medicine at Dartmouth, Hanover, NH USA

**Keywords:** Bladder Cancer, TWAS, GWAS, Case-control, Cancer, Genetics, Risk factors

## Abstract

Genome-wide association studies (GWAS) have detected several susceptibility variants for urinary bladder cancer, but how gene regulation affects disease development remains unclear. To extend GWAS findings, we conducted a transcriptome-wide association study (TWAS) using PrediXcan to predict gene expression levels in whole blood using genome-wide genotype data for 6180 bladder cancer cases and 5699 controls included in the database of Genotypes and Phenotypes (dbGaP). Logistic regression was used to estimate adjusted gene-level odds ratios (OR) per 1-standard deviation higher expression with 95% confidence intervals (CI) for bladder cancer risk. We further assessed associations for individual single-nucleotide polymorphisms (SNPs) used to predict expression levels and proximal loci for genes identified in gene-level analyses with false-discovery rate (FDR) correction. TWAS identified four genes for which expression levels were associated with bladder cancer risk: *SLC39A3* (OR = 0.91, CI = 0.87–0.95, FDR = 0.015), *ZNF737* (OR = 0.91, CI = 0.88–0.95, FDR = 0.016), *FAM53A* (OR = 1.09, CI = 1.05–1.14, FDR = 0.022), and *PPP1R2* (OR = 1.09, CI = 1.05–1.13, FDR = 0.049). Findings from this TWAS enhance our understanding of how genetically-regulated gene expression affects bladder cancer development and point to potential prevention and treatment targets.

**Supplementary Information:**

The online version contains supplementary material available at 10.1038/s41598-025-85565-3.

## Introduction

About 82,000 new cases of urinary bladder cancer are diagnosed annually in the United States^1^. Given its high recurrence rate and costly therapy, bladder cancer carries a considerable morbidity and financial burden^2,3^. Genome-wide association studies (GWAS) have detected several common genetic variants associated with bladder cancer risk^4–7^, yet our understanding of the inherited susceptibility remains incomplete, currently explaining far less of the heritability estimated from twin studies^8,9^. Many GWAS-identified risk variants are located in non-coding regions, making it difficult to determine their function in disease development^10–12^. Additionally, GWAS can have a high multiple-testing burden, requiring large sample sizes to detect modest effect sizes, and associations may be detected only for genotyped single nucleotide polymorphisms (SNPs) correlated with causal variants^13^.

Transcriptome-wide association studies (TWAS) have become a valuable way to investigate disease associations with gene expression^10,14^. TWAS leverages reference data on tissue-specific expression quantitative trait loci (eQTLs) to predict gene expression levels using germline genotype data^10,15^. TWAS offers some advantages over GWAS. Focusing on gene-level associations can lower the multiple-testing burden, minimize the influence of correlated variants, and improve statistical power by aggregating eQTL information^16–18^. The tissue-specificity of TWAS can contribute to distinct insights into biological mechanisms. Koutros et al.^19^ conducted a multi-tissue TWAS based on up to 48 tissue types using bladder cancer cases available from The Cancer Genome Atlas (TCGA) and controls from the Genotype-Tissue Expression (GTEx) project. Multi-tissue approaches may be justified because large repositories of gene expression data collected from normal bladder tissue are lacking. GTEx, for example, currently includes eQTL data for only a small number (*n* = 21) of normal bladder tissue samples. However, aggregating expression data from dozens of tissue types unrelated to bladder physiology likely identifies highly non-specific eQTLs and may dilute signals apparent only in the target tissue. In contrast, we chose to focus on circulating expression data exclusively. The GTEx eQTL database includes a relatively large number (*n* = 670) of whole blood samples, which is sufficient for robust TWAS inference, and this approach may inform potential targets for blood-based cancer screening tests currently under development^20^. We utilized PrediXcan, a popular TWAS method, to predict gene expression levels in whole blood using genome-wide genotype data from several completed bladder cancer case-control studies^4,6,21–23^.

## Results

**Fig. 1:**
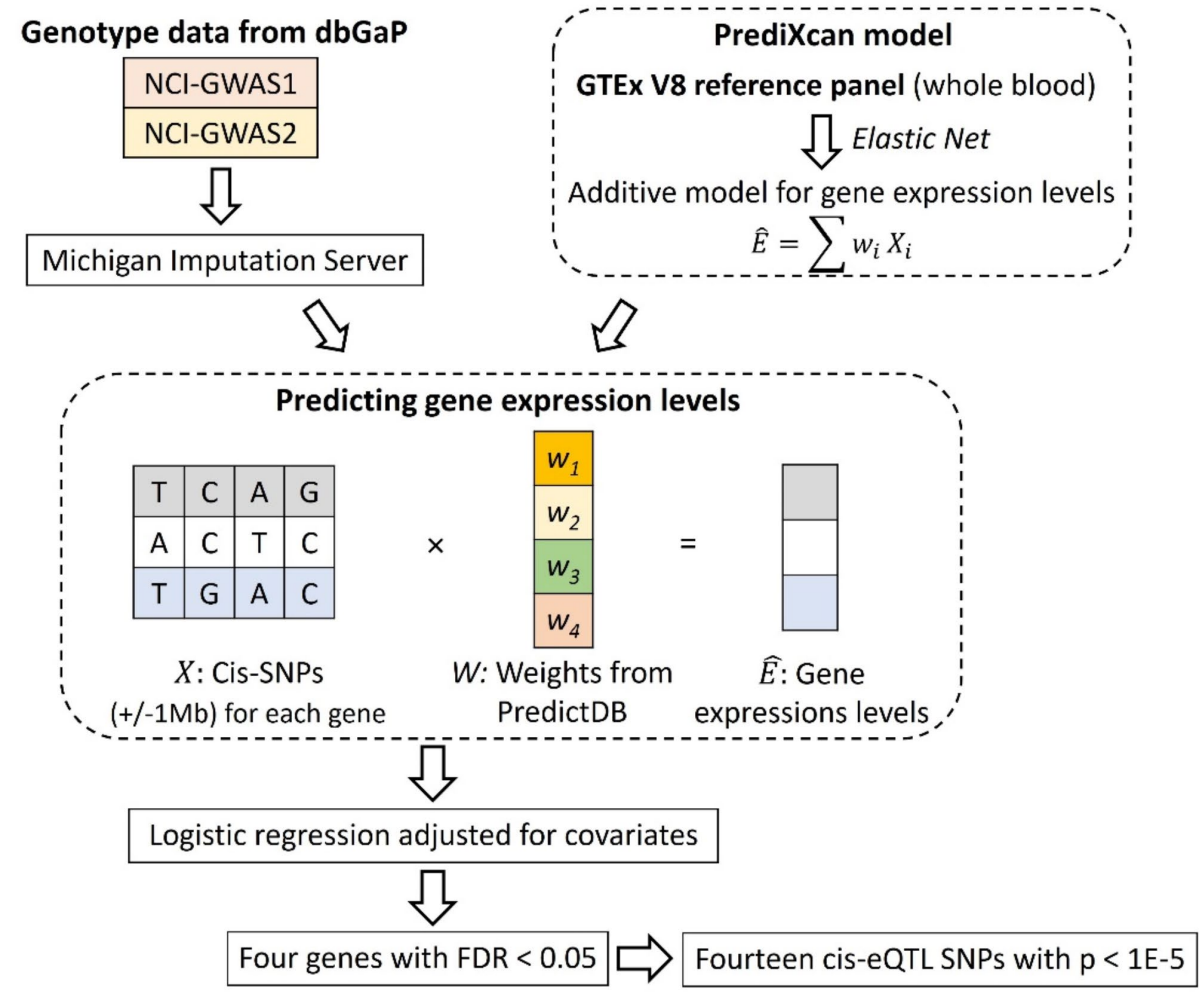
The TWAS was performed using genotype data from a two-phase GWAS funded by the National Cancer Institute (NCI) comprised of 6180 bladder cancer cases and 5699 controls (dbGaP study accession number phs000346.v2.p2). After genotype imputation and QC filtering, a total of 5403 cases and 4672 controls were included in logistic regression models that adjusted for gender, age, and the first three principal components of genetic ancestry.

After quality control (QC), 5403 bladder cancer cases and 4672 controls were included. We predicted cis-eQTL-regulated gene expression levels of 7233 genes from 171,054 SNPs after QC filtering (Fig. 1).

**Fig. 2:**
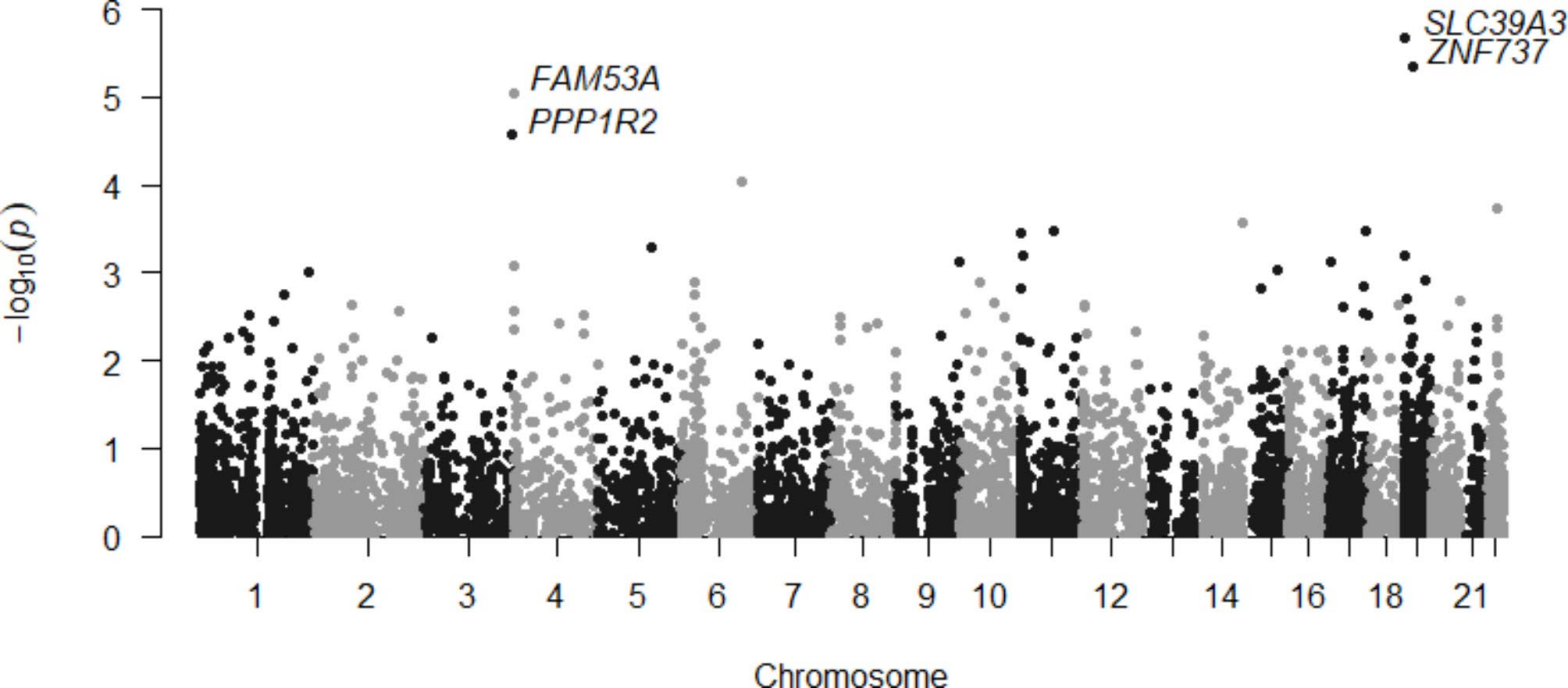
Manhattan plot of TWAS associations between predicted gene expression and bladder cancer risk. The four labeled genes were statistically significant with FDR correction.

A total of four genes (*SLC39A3* on 19p13.3, *ZNF737* on 19p12, *FAM53A* on 4p16.3, and *PPP1R2* on 3q29) were identified to be associated with bladder cancer risk with FDR < 0.05 (Table 1; Fig. 2). Higher whole blood expression of *FAM53A* and *PPP1R2* was associated with higher odds of bladder cancer, while higher expression of *SLC39A3* and *ZNF737* was associated with lower odds of bladder cancer. We did not find evidence of heterogeneity according to gender and age (Supplemental Fig. 1).

**Table 1:** Predicted gene expression levels identified by TWAS with an FDR-adjusted P value < 0.05.

Gene	Location	No. of SNPs^a^	OR (95% CI)^b^	*P* value	FDR-adjusted *P* value
*SLC39A3*	19p13.3	7	0.909 (0.873, 0.945)	2.1E−6	0.015
*ZNF737*	19p12	12	0.911 (0.876, 0.948)	4.4E−6	0.016
*FAM53A*	4p16.3	16	1.095 (1.052, 1.139)	8.9E−6	0.022
*PPP1R2*	3q29	9	1.089 (1.047, 1.133)	2.7E−5	0.049

^a^Number of SNPs used to predict gene expression in whole blood. Of the fifteen SNPs used to predict *ZNF737*, three were unavailable in the post-QC imputed genotype data, while twelve were available. All SNPs for *SLC39A3*, *FAM53A*, and *PPP1R2* were available.

^b^Expression levels were standardized when calculating the odds ratio (OR). A one-unit increase in expression levels corresponds to a one-standard deviation change before standardization.

In single-SNP analyses of 44 cis-eQTLs variants used by PrediXcan to predict gene expression for *SLC39A3*,* ZNF737*,* FAM53A*, and *PPP1R2*, we identified 14 SNPs associated with bladder cancer at *p* < 1.0E–5 (Table 2; Supplementary Fig. 2)^24^. No SNPs of *ZNF737* reached this threshold and no SNPs near any of the four genes reached the genome-wide statistical significance threshold of *p* < 5.0E–8.

**Table 2:** Fourteen cis-eQTL SNPs of the TWAS-identified genes were associated with bladder cancer risk (*p* < 1.0E−5).

SNP	Alleles^a^	Position^b^	*P* value	OR (95% CI)^c^
*SLC39A3* (*p* = 2.1E−6)				
rs759071	A/G	Chr19:2728579	1.2E−6	0.85 (0.80, 0.91)
rs10407667	C/T	Chr19:2731529	1.6E−6	0.86 (0.80, 0.91)
rs4806874	A/G	Chr19:2738354	5.9E−6	0.87 (0.81, 0.92)
rs10415622	A/G	Chr19:2739698	6.1E−6	0.87 (0.81, 0.92)
*FAM53A* (*p* = 8.9E−6)				
rs798726	C/T	Chr4:1683484	4.9E−6	1.18 (1.10, 1.26)
rs798756	C/T	Chr4:1705720	1.7E−6	1.18 (1.10, 1.27)
rs798741	G/A	Chr4:1710686	1.6E−6	1.18 (1.10, 1.27)
rs798744	A/C	Chr4:1712957	1.6E−6	1.18 (1.11, 1.27)
rs2854918	C/T	Chr4:1714966	1.0E−6	1.15 (1.09, 1.22)
rs798763	C/T	Chr4:1729894	3.8E−6	1.15 (1.08, 1.21)
rs2166580	A/G	Chr4:1741442	4.0E−6	1.18 (1.10, 1.26)
*PPP1R2* (*p* = 2.7E−5)				
rs34950021	C/T	Chr3:195511759	1.7E−8	1.20 (1.12, 1.27)
rs1136	T/G	Chr3:195515796	2.1E−8	1.19 (1.12, 1.27)
rs35281308	A/C	Chr3:195541400	1.7E−8	1.20 (1.12, 1.27)

^a^Major allele/Minor allele; ^b^genomic positions for GRCh38; ^c^Odds ratio (OR) of bladder cancer for the minor allele compared with the major allele (reference group).

**Fig. 3:**
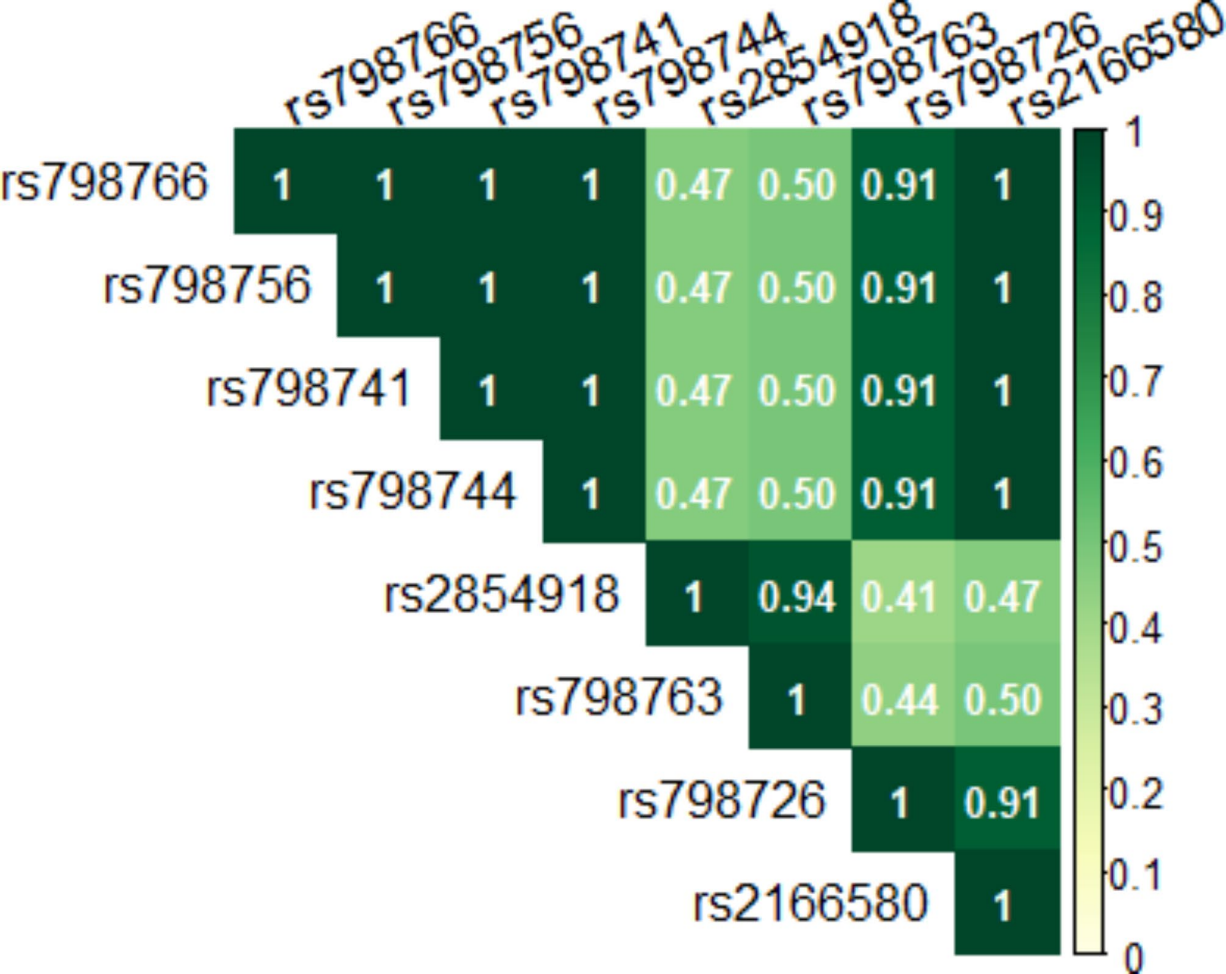
Heatmap of pairwise squared correlations (linkage disequilibrium) of SNPs near *FAM53A* (4p16.3) in the 1000 Genomes CEU population. rs798766 is a GWAS-identified bladder cancer risk SNP and the other SNPs are cis-eQTLs for *FAM53A* in whole blood found to be associated with bladder cancer risk in our TWAS.

GWAS-loci mapping analyses revealed a GWAS-identified bladder cancer risk SNP near those used to predict expression of *FAM53A* (rs798766 located in *TACC3* near *FGFR3*)^4–6^. Five of the *FAM53A* SNPs listed in Table 2 are highly correlated (r^2^ > 0.9) with rs798766 in the 1000 Genomes CEU population: rs798726, rs798741, rs798744, rs798756, and rs2166580 (Fig. 3). We did not identify any GWAS-identified bladder cancer risk SNPs located within 1 Mb of *SLC39A3*, *ZNF737*, and *PPP1R2*.

## Discussion

We performed a TWAS of bladder cancer risk among individuals of European descent leveraging publicly available genotype and transcriptome data, identifying four genes (*SLC39A3*,* ZNF737*,* FAM53A*, and *PPP1R2*) with genetically predicted expression levels in whole blood associated with bladder cancer risk. A known GWAS-identified bladder cancer susceptibility SNP, rs798766 of *TACC3* (near *FGFR3*), was among SNPs associated with *FAM53A* expression in whole blood. Our findings reveal a potential role of blood expression-related genetics in bladder cancer development.

We identified a novel association between lower circulating expression of *SLC39A3* and bladder cancer risk. *SLC39A3* is a member of the solute carrier family 39 of zinc transporters that function in the cellular homeostasis of zinc^25^, an essential trace element involved in multiple physiological processes, including immune function and cellular proliferation^25,26^. SLC39 family members are Zrt, Irt-like Proteins (ZIP) that function to increase cytosolic zinc concentrations^25–27^. Common SNPs of genes in the SLC39 family have been detected to be associated with bladder cancer risk. Karagas et al. reported a higher risk of bladder cancer for carriers of the C allele of a missense variant of *SLC39A2* (rs2234636), specifically among those with high toenail arsenic concentrations^28^. A candidate gene study by Wu et al. found that four SNPs of *SLC39A11* were associated with bladder cancer risk^29^. Although our study did not identify other SLC39 family genes associated with bladder cancer risk, previous studies of tumor expression have suggested that several SLC39 family genes may be involved in bladder cancer prognosis. For example, a pan-cancer TCGA analysis found that tumor overexpression of *SLC39A3*, *SLC39A5*, and *SLC39A11* was associated with better overall survival after bladder cancer diagnosis, whereas tumor overexpression of *SLC39A2*, *SLC39A8*, *SLC39A9*, and *SLC39A14* was associated with poorer survival^30^.

We also found an inverse association between whole blood expression of *ZNF737* and bladder cancer risk. *ZNF737* is a member of the large family of zinc finger proteins (ZNFs), transcription factors frequently studied in cancer^31,32^. Numerous ZNF family genes have been documented to function as tumor promoters or suppressors across different cancer types^31^. *ZNF737* itself is not well characterized. Our finding of higher *ZNF737* expression in association with lower bladder cancer risk is consistent with tumor suppressive activity previously documented for *ZNF750* in esophageal squamous cell carcinoma^33^, *ZNF24* in breast cancer^34^, and *ZNF185* in prostate cancer^35^.

Higher whole blood expression of *FAM53A*, located on 4p16.3, was associated with higher bladder cancer risk. This locus also reached statistical significance in the multi-tissue bladder cancer TWAS conducted by Koutros et al.^19^, but they did not report the direction of association or indicate whether the association was tissue-specific. Among the SNPs we used to predict *FAM53A* expression, four are in perfect linkage disequilibrium with rs798766 (r^2^ = 1 in the 1000 Genomes CEU population), a known GWAS-identified bladder cancer risk SNP^5,36^. Consistent with GWAS findings for rs798766, the minor allele of these SNPs was associated with a higher bladder cancer risk in our data. Previous pre-clinical studies have linked higher expression of neighboring genes to *FAM53A* on 4p16.3, including *TACC3* and *FGFR3*, to bladder cancer development and progression^37,38^. In particular, *FGFR3* mutations and fusions have been well-characterized in bladder tumors^39,40^. Additional research is needed to understand whether circulating *FAM53A* expression could be a marker for 4p16.3 rearrangements and subsequent prognosis.

Our TWAS also suggests that higher circulating *PPP1R2* expression is associated with higher bladder cancer risk. *PPP1R2* encodes one of the regulatory (inhibitory) subunits of protein phosphatase 1 (PP1), which plays a critical role in glycogen metabolism and several basic cellular functions^41–43^. PP1 inhibitors are thought to counteract the tumor-suppressive function of retinoblastoma protein (pRb)^44,45^. This is generally consistent with our observation of an association between higher expression of an inhibitory subunit and higher cancer risk. *PPP1R2* is expressed in many human cancer cell lines^46^, indicating involvement in fundamental oncogenic processes. Studies of the role of *PPP1R2* in bladder cancer specifically, however, are lacking.

This study has some limitations, including those that are inherent to all TWAS^10,14^. We used genotype data to predict gene expression in whole blood rather than in bladder tissue because the sample size of bladder tissue in GTEx is small (*n* = 21), greatly limiting its utility as a TWAS reference panel. TWAS findings based on expression data from normal bladder tissue and bladder tumor tissue, when available, may be interpreted more directly in the context of what is known about organ physiology or tumor environment^14^. On the other hand, TWAS findings based on the circulating transcriptome may be useful in suggesting new targets for emerging blood-based early cancer detection tools.

The dbGaP release did not include information on tumor subtypes or histologies, preventing us from distinguishing muscle-invasive from non-muscle-invasive bladder cancer and urothelial from non-urothelial cancers. We expect some of the associations we identified may be specific to the incidence of early-stage, low-grade disease and thus potentially more useful targets for early detection. Several previous GWAS of bladder cancer risk have adjusted for smoking status^4,6,21^, one of the strongest disease risk factors^47^, but this covariate was unavailable to us. It seems unlikely that additionally adjusting for smoking would have a major impact on our results given that the genes we identified are not among those found to be associated with smoking behaviors or nicotine metabolism^48,49^.

After genotype imputation and QC, the bladder cancer GWAS dataset included 96.6% of the cis-eQTL SNPs in the version of PredictDB for the PrediXcan elastic net models. We did not search for correlated SNPs to use as proxies and thus may be missing signals for some genes. However, it appears that most excluded SNPs were rare or otherwise failed our QC filtering. Single-SNP analyses that we performed secondary to gene-level analyses for genes that met an FDR threshold are reported without multiple comparison correction. Although no single-SNP association reached genome-wide statistical significance, we evaluated only 44 SNPs in total for these analyses, which may not warrant such a stringent threshold to prevent false positives. We included only individuals of European ancestry in our TWAS, potentially limiting generalizability of our findings to other populations. Future studies of larger size should include genotype and transcriptome data from other ancestral populations^50^.

## Materials and methods

### Genotype data and study populations

Genome-wide genotype data for 6180 bladder cancer (in situ and invasive) cases and 5699 controls were ascertained from a two-phase multi-center GWAS of bladder cancer risk accessed from the database of Genotypes and Phenotypes (dbGaP); study accession number phs000346.v2.p2)^4,6,21–23^. Phase I samples (NCI-GWAS1) included participants from the Spanish Bladder Cancer Study (SBCS), New England Bladder Cancer Study - Maine and Vermont (NEBCS-ME/VT), Alpha-Tocopherol, Beta-Carotene Cancer Prevention Study (ATBC), Prostate, Lung, Colorectal and Ovarian Cancer Screening Trial (PLCO), and American Cancer Society Cancer Prevention Study II Nutrition Cohort (CPS-II). Phase II samples (NCI-GWAS2) included participants from the New England Bladder Cancer Study—New Hampshire (NEBCS-NH), Los Angeles Bladder Cancer Study (LABCS), French Center for Research on Prostate Diseases (CeRePP), French Bladder Study (FBCS), Brescia Bladder Cancer Study (BBCS), European Prospective Investigation Into Cancer and Nutrition Study (EPIC), Health Professionals Follow-up Study (HPFS), Women’s Health Initiative (WHI), and the Nurses’ Health Study (NHS)^4,6,21^. A summary of the number of SNPs, genotyping platforms used, and the number of bladder cancer cases and controls from included studies is provided in Supplementary Table 1.

### Quality control and genotype imputation

We conducted pre- and post-imputation genotype quality control (QC) using PLINK^51,52^ following guidelines by Chia et al.^53^. Pre-imputation QC was implemented separately for each genotyping platform. At the individual level, we excluded individuals with more than 5% missing genotypes, with genetic heterozygosity exceeding an F-coefficient threshold (F > 0.15 or F < -0.15), and whose first or second principal components of genetic ancestry were beyond six standard errors from the mean for Europeans using ancestral references from the HapMap 3 Genome Reference Panel^54^. At the SNP level, we removed variants with minor allele frequency (MAF) less than 5%, with more than 5% missing genotypes across individuals, and with evidence of deviation from Hardy-Weinberg equilibrium (*p* < 1.0E−3). We also performed a case/control nonrandom missingness test to exclude SNPs with evidence of missingness differences between cases and controls (*p* < 1.0E−4). Genotype imputation was conducted using the Michigan Imputation Server^55^, employing the Minimac4 algorithm and the 1000 Genome Project Phase 3 version 5 reference panel^56,57^. Phasing was performed using Eagle v2.4. We retained potentially related individuals (pairwise expected shared genotypes ≥ 0.2) for imputation but excluded one individual from each pair for association analyses. Post-imputation QC retained only SNPs with an imputation accuracy R^2^ ≥ 0.3.

### Transcriptome-wide association study

We used PrediXcan^58^ to predict gene expression levels in whole blood for bladder cancer cases and controls from the imputed SNPs that passed QC filtering. PrediXcan trains a prediction model using reference datasets comprising both genome-wide germline genotype and tissue-specific transcriptome data, permitting estimation of eQTL-regulated gene expression levels in an external genotyped dataset without requiring transcriptome data from the same samples. Elastic net model weights were ascertained from PredictDB, a publicly available database that contains pre-computed PrediXcan weights for 48 tissue types from the Genotype-Tissue Expression (GTEx) project^58–60^. We used the GTEx version 8 reference transcriptome data for whole blood and aligned both the GTEx and dbGaP bladder cancer case-control genotype data to the GRCh38 genome assembly^61,62^. All predicted gene expression levels were standardized by subtracting the mean and dividing by the standard deviation before regression analysis to reduce heterogeneity in expression scales.

Logistic regression was used to estimate odds ratios (OR) with 95% confidence intervals (CI) for the association of gene expression with bladder cancer case-control status adjusting for gender, age, and the first three principal components of genetic ancestry estimated from a panel of 2,318 ancestry-informative SNPs^63^. To account for multiple comparisons, a false-discovery rate (FDR) threshold of 0.05 was employed to define statistical significance. Stratified analyses according to gender and age groups (≤ 50, 50–60, 60–70, > 70 years) were conducted given the substantial disparity in disease risk for these groups^64,65^. Association analyses were performed using R^66^; all P values are two-sided.

We further investigated genes found to be associated with bladder cancer risk at the FDR threshold by performing single-SNP analyses focused on the set of variants used to predict gene expression levels with a logistic regression model similar to that used in gene-level analyses. A threshold of *p* < 5.0E−8 was used to define genome-wide statistical significance. SNPs with *p* < 1.0E−5 were also reported. We mapped SNPs within a 1 Mb region upstream or downstream of the TWAS-identified genes with a listing of GWAS-identified bladder cancer risk variants reported in at least two previous GWAS studies for bladder cancer risk included in the NHGRI-EBI GWAS Catalog^7,67^. Squared correlations (linkage disequilibrium) between GWAS-identified SNPs and our TWAS-identified SNPs were calculated using the Ensembl Linkage Disequilibrium Calculator^68^ for the Northern and Western Europeans from Utah (CEU) reference population.

## Electronic supplementary material

Below is the link to the electronic supplementary material.

Supplementary Material 1

## Data Availability

All human data used in this study are de-identified and publicly available upon request and approval through dbGaP (accession number phs000346.v2.p2).
