# Supplementary material for: Transcriptome-wide association study identifies genes associated with bladder cancer risk
Source: Sci Rep. 2025 Jan 9;15:1390. doi: 10.1038/s41598-025-85565-3 (PMC11718161; doi:10.1038/s41598-025-85565-3)
Supplement: Supplementary file 1 — Supplementary Material 1 [file 41598_2025_85565_MOESM1_ESM.docx]

Supplementary Information

**Supplementary Table1.** Characteristics of the genome-wide genotype data for bladder cancer cases and controls accessed from dbGaP (phs000346.v2.p2).

| **Genotyping Platform^a^** | **SNP Number^b^** | **Cases^b^** | **Controls^b^** | **Study^c^** |
| --- | --- | --- | --- | --- |
| Human610_Quadv1_B | 620,901 | 2,485 | 2,273 | NEBCS-ME/VT |
| Human1Mv1_C | 1,072,820 | 1,145 | 1,083 | SBCS |
| HumanHap550v3.0 | 561,466 | 37 | 825 | ATBC, CPS-II or PLCO |
| HumanHap300v1.1 | 317,503 | 31 | 1,150 | ATBC, CPS-II or PLCO |
| HumanHap240S_A | 243,991 | 31 | 1,147 | ATBC, CPS-II or PLCO |
| Human660W-Quad_v1_A | 716,385 | 2,482 | 371 | NCI-GWAS2 |

^a^ All the platforms are Illumina genotyping arrays.

^b^ The number of SNPs, bladder cancer cases and controls before genotype imputation and quality control filtering.

^c^ NEBCS-ME/VT: New England Bladder Cancer Study - Maine and Vermont; SBCS: Spanish Bladder Cancer Study; ATBC: Alpha-Tocopherol, Beta-Carotene Cancer Prevention Study; CPS-II: American Cancer Society Cancer Prevention Study II Nutrition Cohort; PLCO: Prostate, Lung, Colorectal and Ovarian Cancer Screening Trial; NCI-GWAS2: Phase II of the dbGaP bladder cancer GWAS study.

**Supplementary Figure 1.** Forest plot of ORs of significant gene expression levels from TWAS. (a) Grouped by gender, (b) grouped by age. Lines represent the 95% CI of the OR.

(a)


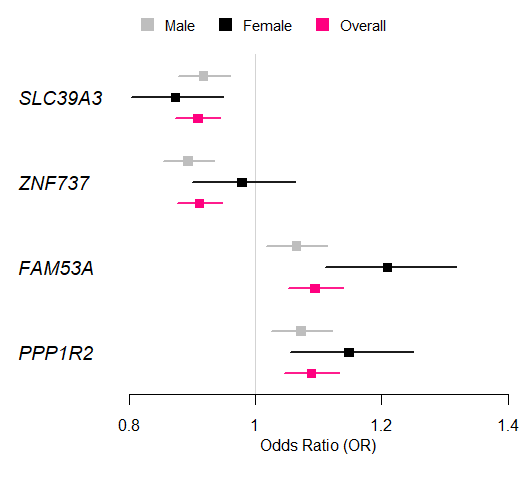


(b)


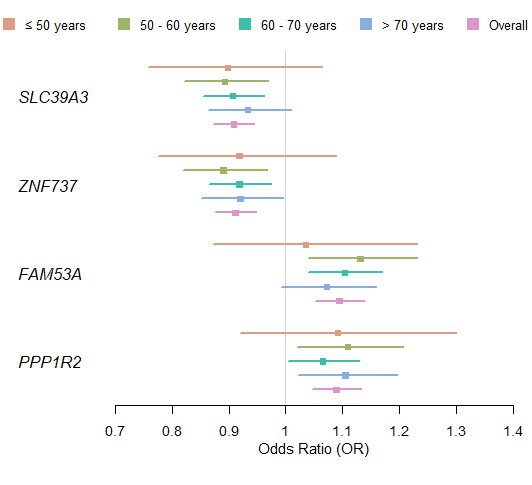


**Supplementary Figure 2.** LocusZoom plots for the association with bladder cancer risk for (a) *SLC39A3*, (b) *ZNF737*, (c) *FAM53A*, (d) *PPP1R2*. SNPs included cis-eQTL SNPs used to predict each gene expression.

Figures were generated using http://locuszoom.org/.

| (a)  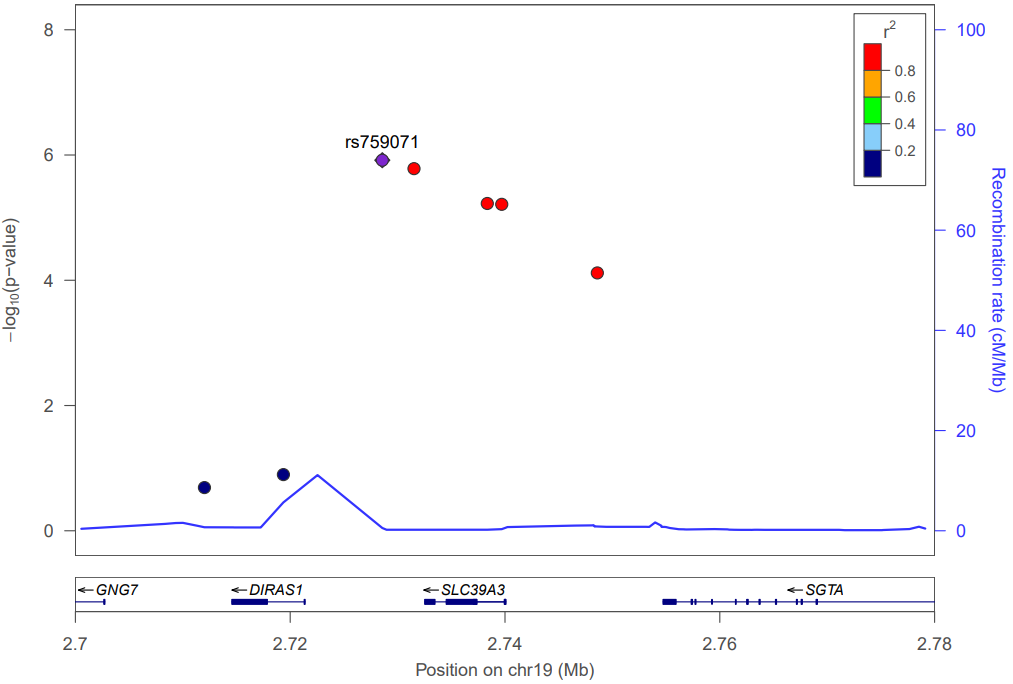 | (b)  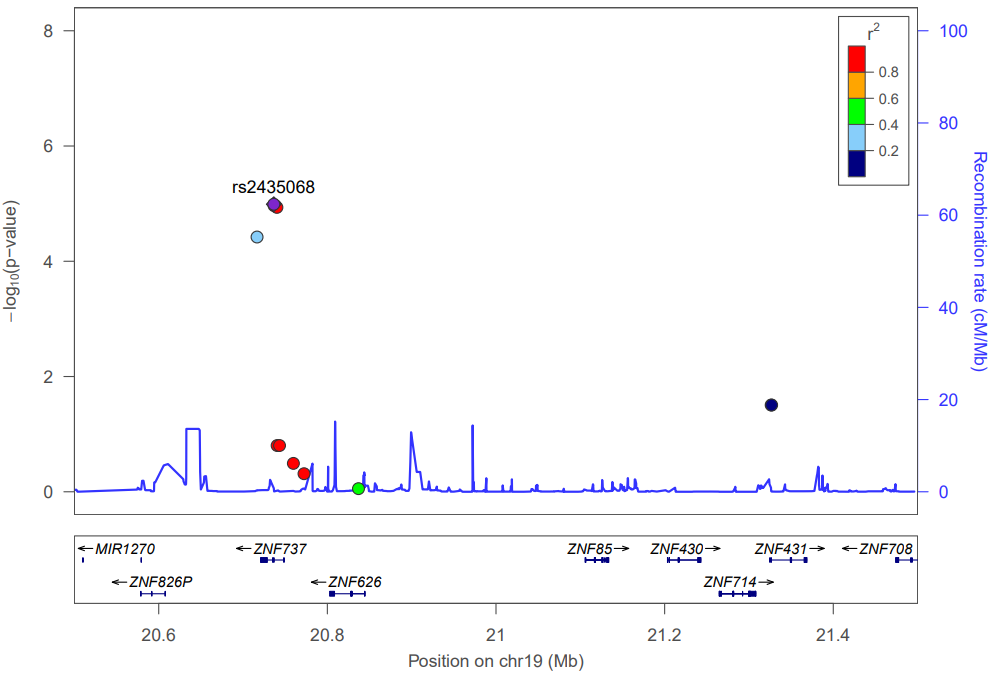 |
| --- | --- |
| (c)  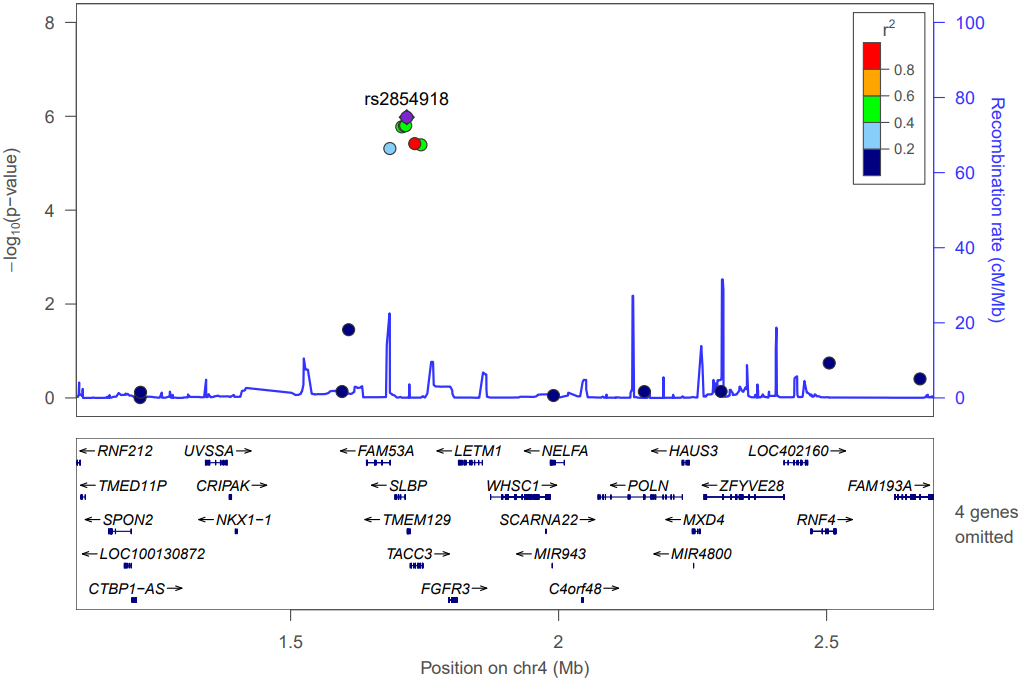 | (d)  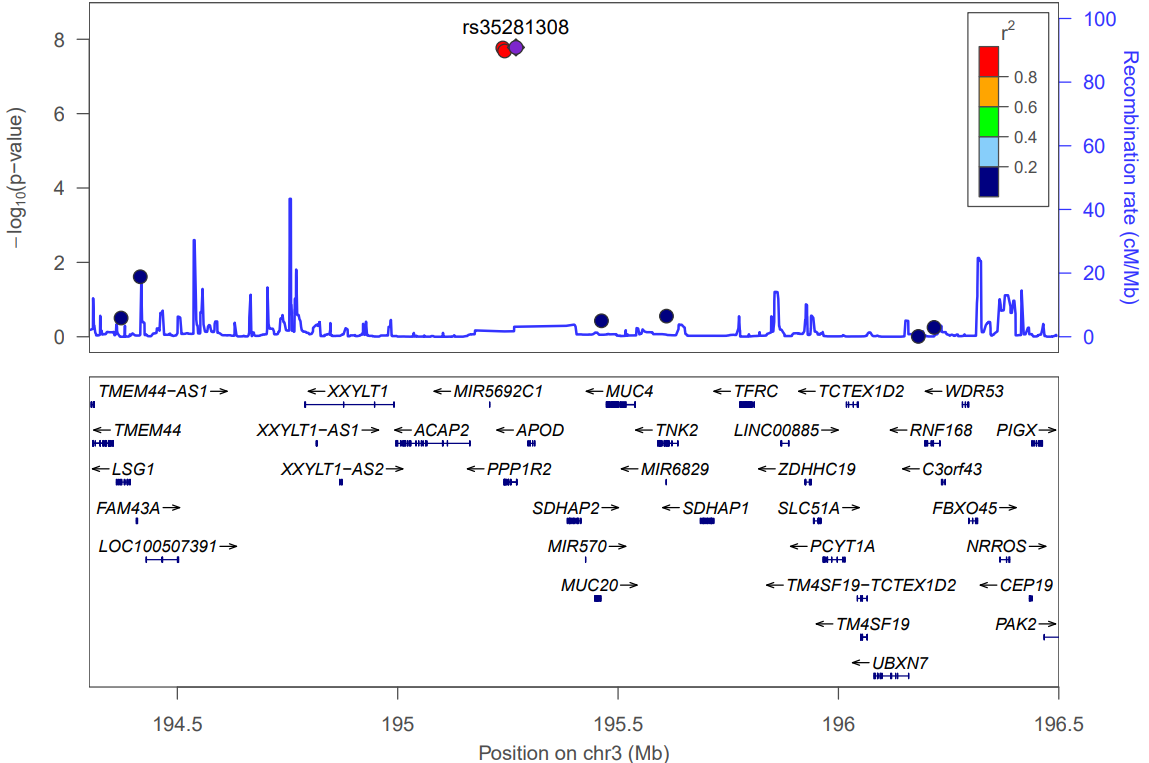 |

**Supplementary Figure 3.** Violin plots of predicted gene expression in whole blood by genotype for the 14 statistically significant cis-eQTL SNPs based on the dbGaP data.

(a) *SLC39A3* and rs759071, (b) *SLC39A3* and rs10407667, (c) *SLC39A3* and rs4806874, (d) *SLC39A3* and rs10415622, (e) *FAM53A* and rs798726, (f) *FAM53A* and rs798756, (g) *FAM53A* and rs798741, (h) *FAM53A* and rs798744, (i) *FAM53A* and rs2854918, (j) *FAM53A* and rs798763, (k) *FAM53A* and rs2166580, (l) *PPP1R2* and rs34950021, (m) *PPP1R2* and rs1136, (n) *PPP1R2* and rs35281308.

| (a)  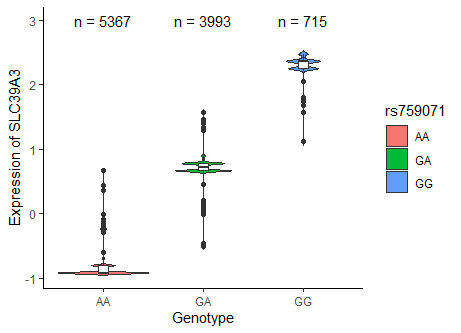 | (b)  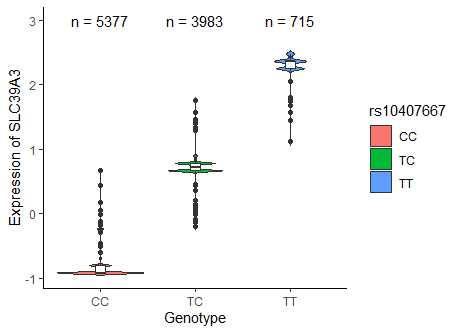 |
| --- | --- |
| (c)  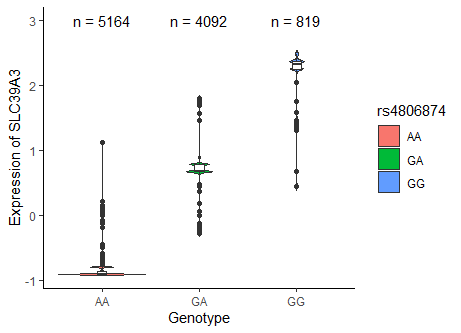 | (d)  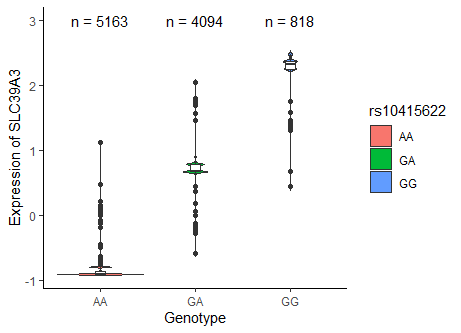 |
| (e)  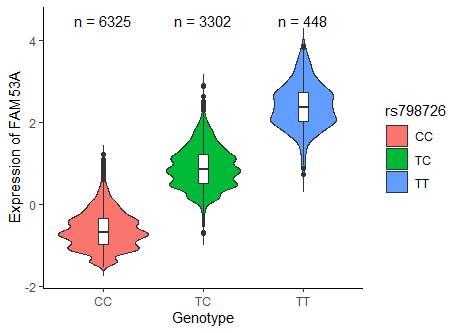 | (f)  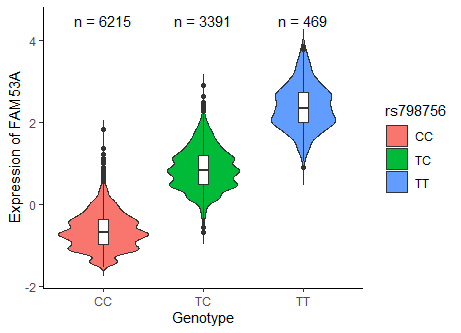 |
| (g)  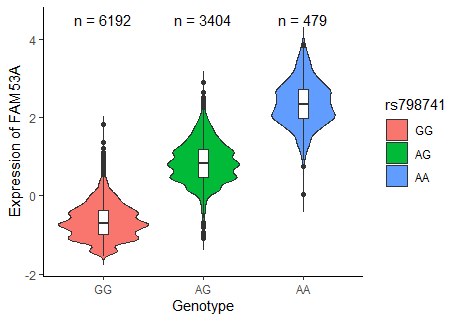 | (h)  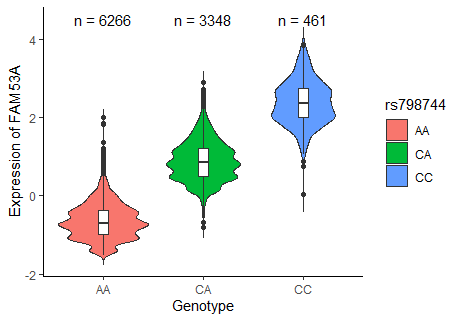 |
| (i)  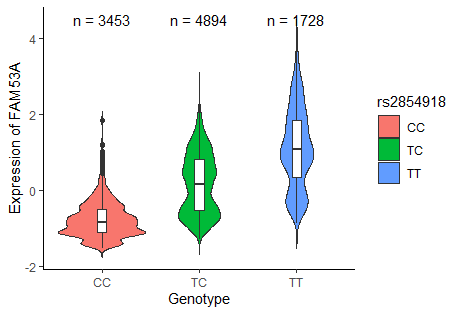 | (j)  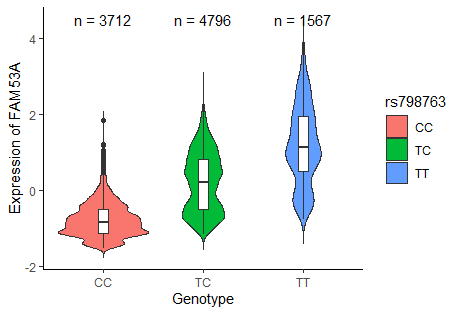 |
| (k)  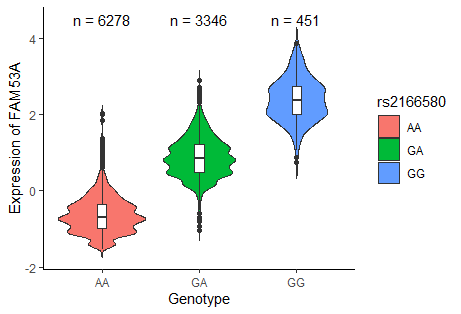 | (l)  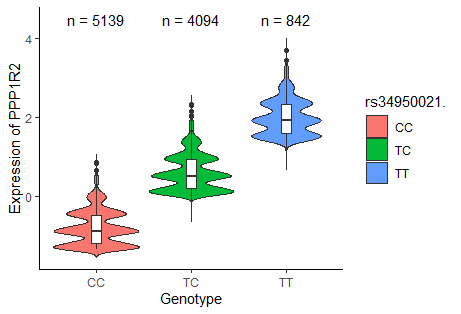 |
| (m)  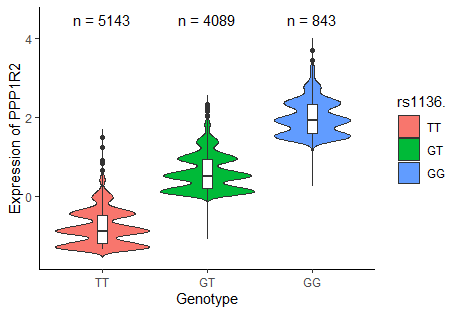 | (n)  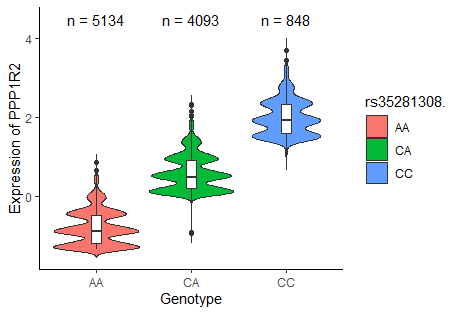 |
